# Age-Associated Differences of Modules and Hubs in Brain Functional Networks

**DOI:** 10.3389/fnagi.2020.607445

**Published:** 2021-01-18

**Authors:** Yinghui Zhang, Yin Wang, Nan Chen, Man Guo, Xiuzhen Wang, Guangcai Chen, Yongchao Li, Lin Yang, Shan Li, Zhijun Yao, Bin Hu

**Affiliations:** ^1^Gansu Provincial Key Laboratory of Wearable Computing, School of Information Science and Engineering, Lanzhou University, Lanzhou, China; ^2^Guangyuan Mental Health Center, Guangyuan, China; ^3^CAS Center for Excellence in Brain Science and Intelligence Technology, Shanghai Institutes for Biological Sciences, Chinese Academy of Sciences, Shanghai, China; ^4^Joint Research Center for Cognitive Neurosensor Technology of Lanzhou University & Institute of Semiconductors, Chinese Academy of Sciences, Lanzhou, China; ^5^Engineering Research Center of Open Source Software and Real-Time System (Lanzhou University), Ministry of Education, Lanzhou, China

**Keywords:** age, resting-state functional magnetic resonance imaging, brain functional network, module, hub

## Abstract

Healthy aging is usually accompanied by changes in the functional modular organization of the human brain, which may result in the decline of cognition and underlying brain dysfunction. However, the relationship between age-related brain functional modular structure differences and cognition remain debatable. In this study, we investigated the age-associated differences of modules and hubs from young, middle and old age groups, using resting-state fMRI data from a large cross-sectional adulthood sample. We first divided the subjects into three age groups and constructed an individual-level network for each subject. Subsequently, a module-guided group-level network construction method was applied to form a weighted network for each group from which functional modules were detected. The intra- and inter-modular connectivities were observed negatively correlated with age. According to the detected modules, we found the number of connector hubs in the young group was more than middle-age and old group, while the quantity of provincial hubs in middle-age group was discovered more than other two groups. Further ROI-wise analysis shows that different hubs have distinct age-associated trajectories of intra- and inter-modular connections, which suggests the different types of topological role transitions in functional networks across age groups. Our results indicated an inverse association between functional segregation/integration with age, which demonstrated age-associated differences in communication effeciency. This study provides a new perspective and useful information to better understand the normal aging of brain networks.

## Introduction

Population aging is a widespread worldwide phenomenon and has also been one of the research hotspots in the neuroimaging field (Ferreira and Busatto, 2013). Aging leads to a large number of psychological, biological, physical, and chemical changes (Huang et al., 2015), resulting in the degradation of working memory, executive function, and processing speed across the lifespan (Bäckman et al., 2006; Raz and Rodrigue, 2006; Bishop et al., 2010; Yao et al., 2012). Growing evidence indicates that age is related to the altered configuration of large-scale functional brain networks, which may have implications for cognitive performance (Onoda et al., 2012; Ng et al., 2016).

In recent years, resting-state functional magnetic resonance imaging (rs-fMRI) has become a powerful tool to explore alterations in the aging brain (Cao et al., 2014; Tian et al., 2016; Chen et al., 2019). A common finding among rs-fMRI studies has indicated that the human brain is functionally organized into an efficient network architecture (Sporns et al., 2005). Studies examining the topology of brain network have indicated several characteristics of an efficient architecture, including the modular architecture and a small number of highly connected hubs (Van Den Heuvel and Pol, 2010; Van den Heuvel and Sporns, 2013a). Aging has been shown to affect the functional modular organization and functional hubs of the human brain (Tomasi and Volkow, 2012; Bertolero et al., 2015; Schlesinger et al., 2017). In the brain functional networks, some subsets of nodes may be highly inter-connected, effectively forming several clustered modules, which represent regions of high correlation in the brain (Moussa et al., 2012). Previous studies have indicated that each module of functional networks is associated with specific cognitive/behavior function (Bertolero et al., 2015). Chen et al. found a decrease in the quantity of modules across age groups, consisting with the evidence of age-related modularity decline (Chen et al., 2019). In a longitudinal study, Chong et al. found that the distinctiveness of modules was negatively correlated with age, especially in higher-order cognitive modules (Chong et al., 2019). Previous research has demonstrated that many hubs with numerous connections disappear while some specific age-associated hubs appear during aging (Simkó et al., 2009).

The brain modular architecture allows for functionally specialized processing (i.e., functional segregation) as well as the integration of information (i.e., functional integration) (Sun et al., 2012). Functional segregation refers to highly clustered connections within modules while functional integration refers to connections between modules that facilitate the integration of information from different modules (Damoiseaux, 2017). Previous works have manifested that age is associated with reduced network segregation (Grady et al., 2016; Damoiseaux, 2017; King et al., 2018). However, some debates exist about the integration of complex brain networks. Previous work has shown that aging is related to the global decrease in integration (Chong et al., 2019; Oschmann and Gawryluk, 2020). On the contrary, some research has found that aging is accompanied by the increase of global measures of integration, i.e., global efficiency (Chan et al., 2014; Yao et al., 2019). Moreover, other studies have declared no age-associated alterations in global efficiency (Cao et al., 2014; Geerligs et al., 2015).

To date, age-related differences in functional modular organization have mainly been inferred from the comparisons of younger vs. older adults. More detailed age information is not considered due to the lack of subdivision of age groups across lifespan, resulting in insufficiently detailed age-related differences in the results. Thus, studies including wide age ranges are needed to fully characterize the aging process. Besides, the difference of modular structure is usually measured by the comparison among average networks formed over large groups. However, the modular structure not only varies across the lifespan but also exhibits individual differences regardless of age (Puxeddu et al., 2020). Therefore, averaging networks across large populations probably results in the loss of essential information. To avoid this, it is necessary to retain the possible unevenness of the network and greatly hold the role played by individuals at the group in the construction of group-level networks.

To solve the aforementioned problems, the present research investigated age-associated differences of modules and hubs using resting-state fMRI data from the Southwest University adult lifespan database (Wei et al., 2018). After dividing the dataset into young, middle, and old age groups, we used a module-guided group-level network construction method to generate weighted group-level networks and explore the modular structure of each group obtained from group-level networks. The current study aims to (1) delineate the age-associated differences of functional modular organization in functional networks from three age groups, and (2) explore the potential driving forces related to aging with clear evidence by using network topological analysis. According to the group-level modular structure, we detected hubs (the nodes that have particularly high connectivity to other nodes) and investigated different age-associated differences of both provincial hubs (the regions that play a highly central role when connecting regions in the same module) and connector hubs (the regions that play a highly central role when connecting different modules), including differences in spatial distribution and quantity. We hypothesize that (1) the age-associated differences of modular organization may be related to specific age-related network integration and segregation; and (2) the spatial distribution and quantity of functional hubs in three groups differs, representing their different age-associated topological roles in functional networks.

## Materials and Methods

### Participants

**Figure 1:**
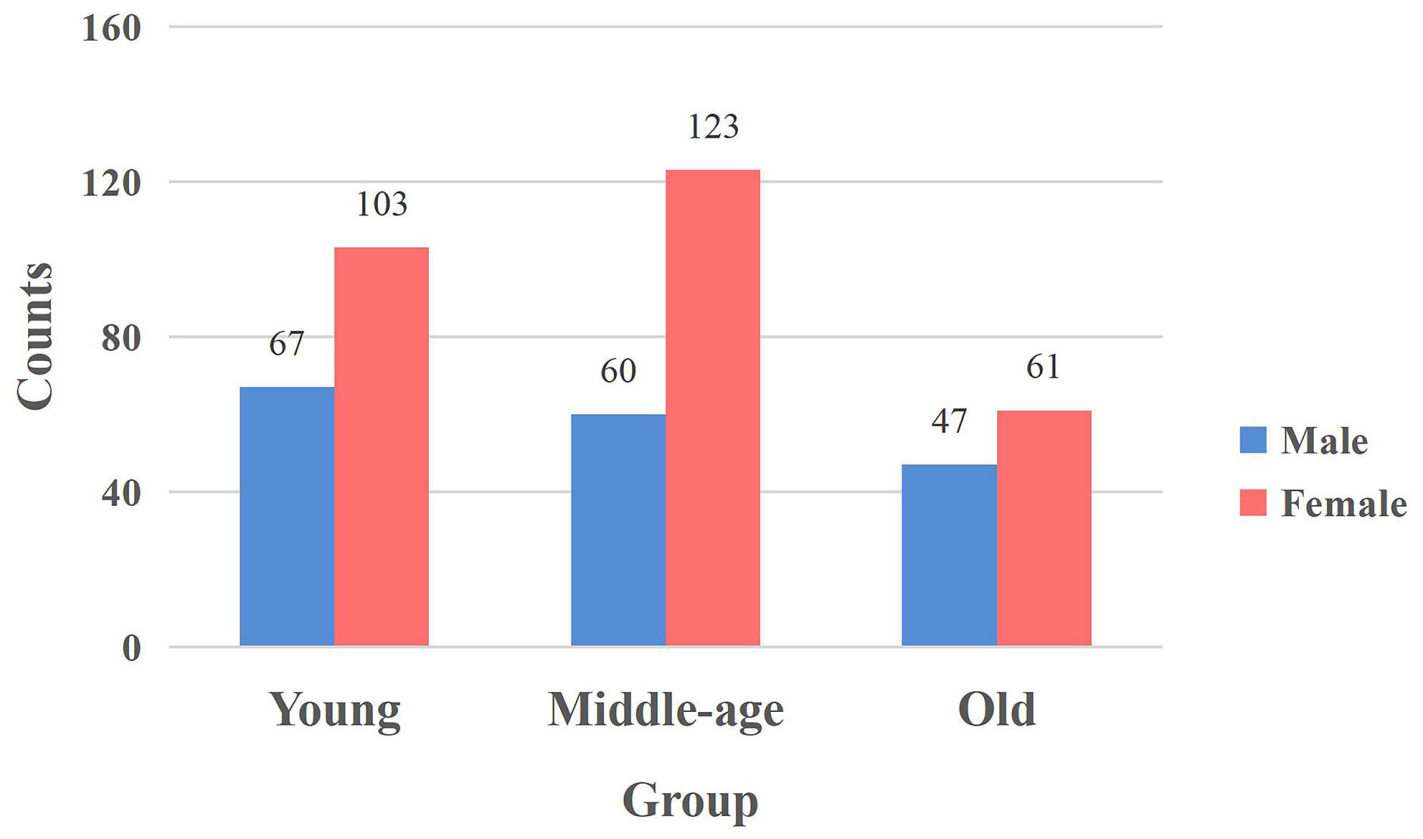
The distribution of males and females in each group. The vertical axis signifies the number of participants, and the horizontal axis represents the age group. The blue bar and red bar represent male and female subjects.

In this work, we used the Southwest University Adult Lifespan Dataset (SALD) (Wei et al., 2018). The dataset collection was approved by the Research Ethics Committee of the Brain Imaging Center of Southwest University. There are no uniform standards of age boundaries for the division of young, middle and old adults. Previous studies applied different age boundaries due to various datasets and research methods and we select the same age boundaries as in previous studies (Sie et al., 2019) due to the use of the same dataset. The dataset included 494 healthy volunteers (187 males and 307 females, aged 19–80) in which 2 participants did not complete the resting-state scanning and 31 participants were excluded due to excessive head motion. The remaining 461 participants were grouped into Young (19–34), Middle (35–59), and Old (60–80) age groups (Sie et al., 2019), with 170, 183, and 108 subjects in each group. Figure 1 showed the distribution of males and females of the groups. Concerning gender, no significant differences were detected between groups using the chi-square test (*p* = 0.161 > 0.05). Each participant gave a written informed consent before the data collection.

### Data Acquisition

All functional images were obtained from a 3.0-T Siemens Trio MRI scanner (Siemens Medical, Erlangen, Germany). The gradient echo-planar imaging (EPI) sequences were used for resting-state fMRI data collection: slices = 32, repetition time (TR)/echo time (TE) = 2,000/30 ms, flip angle (FA) = 90°, field of view (FOV) = 220 *mm* × 220 *mm*, thickness = 3 mm, slice gap = 1 mm, and voxel size = 3.4 × 3.4 × 4 *mm*^3^. During the resting-state MRI scan, participants were asked to lie down, close their eyes, and rest without thinking about any specific thing but to refrain from falling asleep. The scan lasted for 484 s and acquired 242 volumes in total for each subject. Besides, structural images were acquired to provide an anatomical reference using the following magnetization-prepared rapid gradient echo (MPRAGE) sequence: slices = 176, TR/TE = 1,900/2.52 ms, inversion time (TI) = 900 ms, FA = 90°, FOV = 256 *mm* × 256 *mm*, thickness = 1 mm, voxel size = 1 × 1 × 1 *mm*^3^ (Sie et al., 2019).

### Imaging Pre-processing

Using Data Processing Assistant Resting-State Fmri Advanced Edition (DPARSFA; http://www.restfmri.net) (Chao-Gan and Yu-Feng, 2010), the resting-state fMRI data were preprocessed. Preprocessing steps included removing the first 10 volumes, slice-timing, head motion correction, and realignment. Participants with the maximum translation > 3 mm, rotation > 3°, and mean frame-wise displacement (FD) > 0.3 mm were excluded (Spreng et al., 2016). Then the functional images were co-registered to the corresponding T1-weighted images and were normalized to Montreal Neurological Institute (MNI) space with a resampling voxel size of 3 × 3 × 3*mm*^3^ resolution (Wang et al., 2020). Resulting images were spatially smoothed using a 6 mm full-width half-maximum (FWHM) Gaussian kernel, followed by temporal band-pass filtering (0.01–0.1 Hz) to reduce low-frequency drift and high-frequency noise (Van Dijk et al., 2010). Given that global signal regression (GSR) can disturb correlation coefficients (mainly the presence of negative correlations) (Murphy et al., 2009; Wang et al., 2014) and physiological noise (part of global signal) in the BOLD signal is found related to age (Makedonov et al., 2013), we applied GSR to reduce the effects of noise differences across age groups on the estimation of correlation coefficient, and restricted our explorations to positive correlation as in previous studies (Cao et al., 2014; Chong et al., 2019). Finally, nuisance signals representing head motion parameters, global signals, white matter and cerebrospinal fluid signals were regressed out from the data.

### Construction of Brain Networks

The human Brainnetome atlas (Fan et al., 2016) was used to parcellate the whole brain into 246 regions. For each subject, the averaged resting-state fMRI time series were exacted in each brain region, and the Pearson correlation was calculated between the time series of 246 regions to obtain the functional connectivity (FC) matrix. Thus, the FC network for each subject was constructed. In prior studies, negative correlations between brain regions were interpreted as an artifact of global signal regression in Fox et al. (2009), Uddin et al. (2009), and Parente et al. (2018). Recently, some researchers found a relationship between anti-correlations and several biological/psychological variables (Whitfield-Gabrieli et al., 2009; Chai et al., 2012; Wong et al., 2012; Keller et al., 2015), but the biological basis to negative correlations is still debatable. Since the biological meaning of negative correlations is unclear and not well-understood (Chai et al., 2012; Murphy and Fox, 2017), we set negative weights to zero and all positive connections were kept (Wen et al., 2019). The weighted individual-level FC networks are the basis for subsequent experiments.

In this study, the method “Module-Guided Group-Level Network Construction” was used to generate group-level weighted networks and detect robust functional network modules for three age groups (Wen et al., 2019), which took advantage of the information of the detected individual-level modular structures and utilized them as the basis to guide the construction of FC network at group-level.

**Figure 2:**
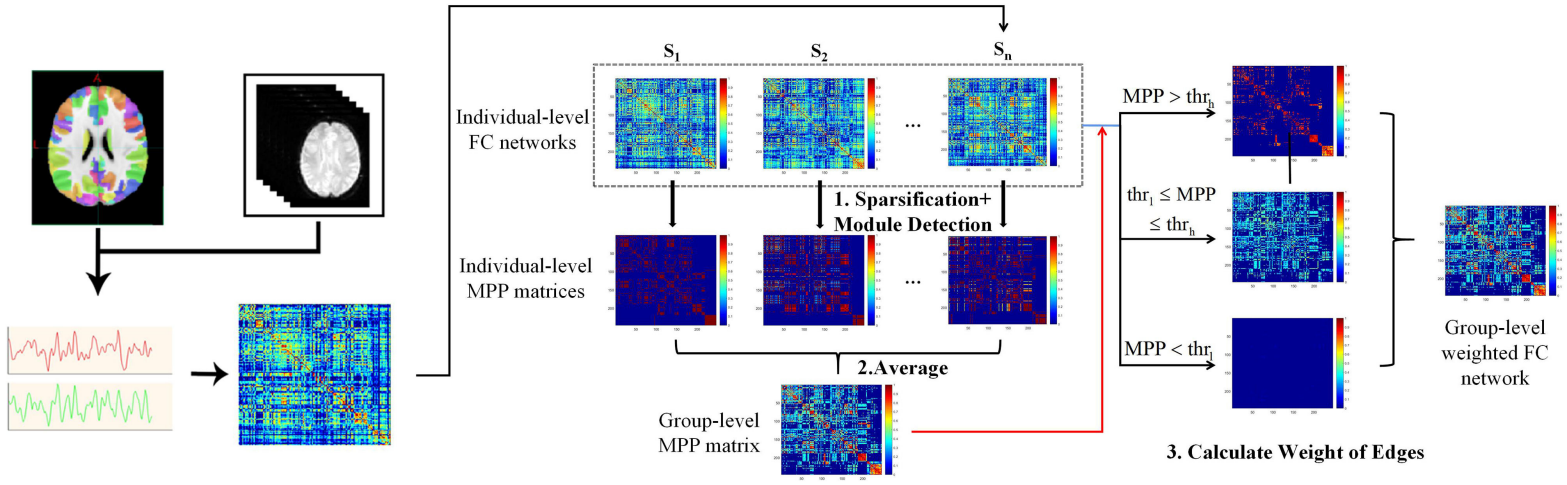
Flowchart of brain network construction, including individual-level FC construction and the module-guided group-level network construction. First, we detected modular partition for individual-level FC networks and converted each modular partition into a matrix. That is, if two nodes are detected in the same module, the weight of the corresponding edge is 1, 0 otherwise. The modular partition matrices of each subject were averaged to obtain the individual-level “modular partition probability” (MPP) matrix, where the weight of one edge represents the probability that the corresponding two nodes assigned to the same module. Second, a group-level MPP matrix representing the module partition probability at group-level was generated by averaging all individual-level MPP matrices in the same group. Third, by combining individual-level FC networks and different weighting methods (depending on the group-level MPP and *thr*_*l*_ / *thr*_*h*_), we finally generated a group-level FC network for the group.

As shown in pipeline overview (Figure 2), the method consists of three steps. Taking one group as an example: First, the top 10% strongest positive connections for each individual-level FC network were kept to remove weak connections and to keep the sparsity of FC networks (Najafi et al., 2016). We then used a complex network analysis toolbox (Radatools, deim.urv.cat/~sergio.gomez/radatools.php) to detect the individual-level modular structure. By using this toolbox, community detection can be implemented by the optimization of modularity in complex networks, which allows to use multiple heuristic algorithms in an iterative manner and output the optimal partition with the highest modularity (Q). The extremal optimization (Duch and Arenas, 2005), tabu search (Arenas et al., 2008), spectral optimization (Newman, 2006), fast algorithm (Newman, 2004) were combined to obtain the optimal partition. However, some heuristics have a stochastic behavior, thus several executions could lead to different optimal partitions. To avoid the stochastic behavior of module detection, we repeated the module detection to each individual-level FC network for 100 times to obtain 100 modular partition results for each participant, though the partitions may be different (Wen et al., 2019). We converted each detected modular partition into a “modular partition matrix.” That is, if two nodes are detected in the same module, the weight of the corresponding edge is 1, 0 otherwise. After that, the 100 modular partition matrices of each subject were averaged to obtain the individual-level “modular partition probability” (MPP) matrix, where the weight of one edge represents the probability that the corresponding two nodes is assigned to the same module. Second, we constructed a group-level MPP matrix representing the module partition probability at group-level by averaging all individual-level MPP matrices in the same group. Similar to individual-level MPP, the edges with higher weight in group-level MPP means the corresponding two nodes are more likely to be assigned to the same module, while the edges with lower weight mean the corresponding two nodes are more likely to be assigned to the different module. In other words, the edges in the group-level MPP with higher weight are more probably strong intra-modular connections, while the ones that have lower weight are more likely to be inter-modular connections. Third, based on the group-level MPP obtained from the second step, we generated a MPP-guided group-level FC network. We used two thresholds (ie., *thr*_*l*_ and *thr*_*h*_) to divide all edges in the group-level MPP matrix into three types. For each type of edge, by combining individual-level FC networks and different weighting methods, we finally generated a group-level FC network for the group by referencing the work of Wen et al. (2019). If the weight of one edge in the group-level MPP is greater than *thr*_*h*_, the final weight of this edge is set to be the average of the five highest weights of all individual-level FC networks in this group on the same edge. Similarly, the final weight of the edge is set to be the average of the five weakest weights on the same edge of individual-level FC networks across all subjects, if the edge in the group-level MPP is lower than *thr*_*l*_. For the edges between *thr*_*l*_ and *thr*_*h*_, we set the final weights to be the average weights of all individual-level FC networks. In this experiment, the *thr*_*l*_ and *thr*_*h*_ were set to 0.1 and 0.5, respectively. For detailed information about *thr*_*i*_ (individual-level network sparsity), *thr*_*l*_ and *thr*_*h*_, please see section Threshold selection.

### Module Detection From Group-Level Network

The modular structures of three MPP-guided group-level FC networks were detected with the same method used for module detection at the individual-level. After repeating module detection to each group-level network for 100 times, a consensus clustering method (Bassett et al., 2013) was used to obtain the group-level consistent modules (for avoiding the stochastic behavior of module detection). Based on the group-level modular structure, we calculated the mean intra- and inter-modular connectivities across all modules for each individual-level FC network in three age groups and evaluated the relationship between age and network integration and separation. The mean intra-modular connectivity was calculated as the average FC strength of all intra-modular connections, which reflected functional segregation. Similarly, the mean inter-modular connectivity reflecting functional integration was measured by the average FC strength of edges across modules.

After obtaining the mean intra- and inter-modular connectivities of each individual-level FC network at all age groups, we applied the general linear model (GLM) (Friston et al., 1994) to describe the age-associated trajectories of intra- and inter-modular connectivities. By using age as an independent variable and the mean intra- and inter-modular connectivities of each subject as dependent variables, the relationship between age and functional segregation and integration of individual FC networks can be evaluated. The threshold for statistical significance was defined as *p* < 0.05.

### Hub Detection and Age-Associated Trajectories of WD and PC

According to the group-level modular structure obtained from section Module detection from group-level network, within-module degree (WD) and participation coefficient (PC) were calculated to evaluate the interactions within and between different brain modules. WD measures the importance of a brain region when connecting other regions belonging to the same module (Guimera and Amaral, 2005). PC quantifies how important a brain region is when linking with different modules (Guimera and Amaral, 2005). Suppose a modular partition is *M* = {*m*_*c*_|*c* = 1, …, *C*}. For a node i in module *m*_*c*_, *WD*_*i*_ and *PC*_*i*_ were defined as:

(1) $$\begin{array}{l} {WD}_{i}=\frac{k_{m_{c}}^{i}-\bar{k_{m_{c}}}}{\delta_{m_{c}}} \end{array}$$

(2) $$\begin{array}{l} {PC}_{i}=1-\overset{C}{\underset{c=1}{\sum }}\left(\frac{k_{m_{c}}^{i}}{k_{i}}\right) \end{array}$$

where $k_{m_{c}}^{i}$ is the total FC strength connected to node i in module *m*_*c*_. $\bar{k_{m_{c}}}$ and δ_*m*_*c*__ are defined as the mean and standard deviation of $k_{m_{c}}^{i}$ across all nodes in module *m*_*c*_. Besides, *k*_*i*_ is the total FC strength of the edges connected to node i.

Hubs are nodes of a network that are particularly highly connected to other nodes (Tang et al., 2019). Hubs are critical for the integration of information and efficient communication in the brain, the disruptions of hub connections are related to many manifestations of brain dysfunction (Bullmore and Sporns, 2012). An important approach to defining network hubs depends on their roles in integrating network modules (Fortunato, 2010). Several studies have shown that hubs could be divided into provincial and connector hubs based on their topological positions in the network measured by WD and PC (Power et al., 2013; Bertolero et al., 2015; Chong et al., 2019). PC divides nodes into two categories—connector nodes with numerous global edges connecting different modules and local nodes with many local edges connecting regions within the module. Connector nodes are considered to integrate information between modules for efficiency information exchange while local nodes integrate information within modules for specialized function (Cole et al., 2013; Power et al., 2013). WD subdivides connector nodes into “satellite connectors” and “connector hubs” that both have high PC, whereas only connector hubs have high WD (greater modular segregation). Similarly, local nodes are subdivided into “peripheral nodes” and “provincial hubs” that both have low PC, in which only provincial hubs have high WD (greater modular integration) (Guimera and Amaral, 2005; Guimera et al., 2005, 2007). Thus, connector hubs should have both high PC and WD, whereas provincial hubs have low PC and high WD (Wen et al., 2019). By setting thresholds for WD and PC (*thr*_*WD*_ and *thr*_*PC*_), we detected hubs and divided them into provincial and connector hubs from three group-level networks. Taking node i for example, if *WD*_*i*_ > *thr*_*WD*_ and *PC*_*i*_ > *thr*_*PC*_, the node is categorized as connector hub, otherwise, if *WD*_*i*_ > *thr*_*WD*_ but *PC*_*i*_ < *thr*_*PC*_, it is identified as a provincial hub. In this experiment results, we only exhibit the hubs when *thr*_*WD*_ was set to 1.0 and *thr*_*PC*_ was set to 0.55. For detailed information about *thr*_*WD*_ and *thr*_*PC*_, see section Threshold Selection.

After detecting hubs for each age group, we selected hub regions detected in the middle-age group as regions of interest (ROIs) and further calculated the WD and PC of the ROIs across all subjects in three groups, for the reason that middle-age is in the transition phase between brain development and aging and can help understand age-related differences. GLM was also applied to characterize age-associated trajectories of WD and PC to investigate the emergence and disappearance of provincial/connector hubs and its possible driving factors. Age was used as an independent variable and the WD and PC of each ROI as dependent variables to describe the age-associated trajectories of WD and PC of these ROIs. The threshold for statistical significance was set as *p* < 0.001 after false discovery rate (FDR) correction.

### Threshold Selection

In the group-level network construction, normalized mutual information (NMI) (Alexander-Bloch et al., 2012) and modularity (Blondel et al., 2008) differences were used as evaluation indexes for the comparison. The NMI and modularity are defined as:

(3) $$\begin{array}{l} \text{NMI}\left(\text{A},\text{B}\right)=\frac{-2\sum_{i=1}^{C_{A}}\sum_{j=1}^{C_{B}}N_{ij}\log\left(\frac{N_{ij}N}{N_{i.}N_{.j}}\right)}{\sum_{i=1}^{C_{A}}N_{i.}\log\left(\frac{N_{i.}}{N}\right)+\sum_{j=1}^{C_{B}}N_{.j}\log\left(\frac{N_{.j}}{N}\right)} \end{array}$$

where A and B are module partitions of two networks, *C*_*A*_ is the number of modules in the partition A, *N*_*ij*_ is the overlap between A's module i and B's module j, N is the total number of nodes, *N*_*i*._ and *N*_.*j*_ represent the total number of nodes in A's module i and in B's module j, respectively. The NMI ranges from 0 to 1, NMI is 0 if the partitions are totally independent, and the value equals to 1 if the partitions are identical (Hinrich et al., 2016).

(4) $$\begin{array}{l} \text{Q}=\frac{1}{2m}\underset{i,j}{\sum }\left[A_{ij}-\frac{k_{i}k_{j}}{2m}\right]\delta \left(c_{i},c_{j}\right) \end{array}$$

where *A*_*ij*_ is FC strength connecting the node i and j, $m=\frac{1}{2}\underset{ij}{\sum }A_{ij}$, *k*_*i*_ represents the sum of FC strength connecting node i, *c*_*j*_ is the module that node j belongs to, δ(*u, v*) equals to 1 if *u* = *v* and 0 otherwise. The modularity (Q) quantifies the strength of segregation into different networks. High Q scores mean highly modular networks, which contain segregated modules and fewer inter-modular connections (Cohen and D'Esposito, 2016; Sporns and Betzel, 2016).

To test the *thr*_*i*_ for individual-level network sparsity, we changed *thr*_*i*_ from 0.05 to 0.15 with 0.01 as an increment. At each setting, we detected the individual-level modular structure and further constructed the group-level FC network for each group. Subsequently, we detected the corresponding group-level modular structure and calculated its similarity to the result with *thr*_*i*_ = 0.1 by using NMI in each group, respectively. As shown in Supplementary Figure 1, the settings of *thr*_*i*_ shows similar group-level modular structures (Wen et al., 2019). The *thr*_*i*_ used in our study was set to 0.1, as in previous studies (Yeo et al., 2014; Najafi et al., 2016; Wen et al., 2019).

To test the *thr*_*l*_ and *thr*_*h*_ for group-level MPP matrices, we respectively set the *thr*_*l*_ from 0.05 to 0.25 and *thr*_*h*_ from 0.45 to 0.65 with a step of 0.05. For different combinations, after re-generating group-level networks for three age groups, the detected consistent modular structures were compared with that constructed with *thr*_*l*_ = 0.1 and *thr*_*h*_ = 0.5 using NMI and Q in each group. The changes induced by *thr*_*l*_ and *thr*_*h*_ are quite small in three age groups (Supplementary Figure 2).

In hub detection, *thr*_*WD*_ and *thr*_*PC*_ have influence on the hub classification. By fixing *thr*_*PC*_ to 0.55 and varying *thr*_*WD*_ from 0.8 to 1.1 with a step of 0.1, the hubs in each group did not change a lot in the spatial distribution or quantity and *thr*_*WD*_ settings of hubs showed similar differences. Similarly, by fixing *thr*_*WD*_ to 1.0 and increasing *thr*_*PC*_ from 0.5 to 0.65 with 0.05 as an increment, the impacts of *thr*_*PC*_ on the hub assessment were also tested. Although the number of connector hubs decreased as *thr*_*PC*_ increased, the setting of *thr*_*PC*_ has no effect on the difference in two types of hubs' quantity between three age groups (Supplementary Figure 3). We thus took the median of the range as thresholds, respectively (Wen et al., 2019).

## Results

### Group-Level Networks and Modular Structures

**Figure 3:**
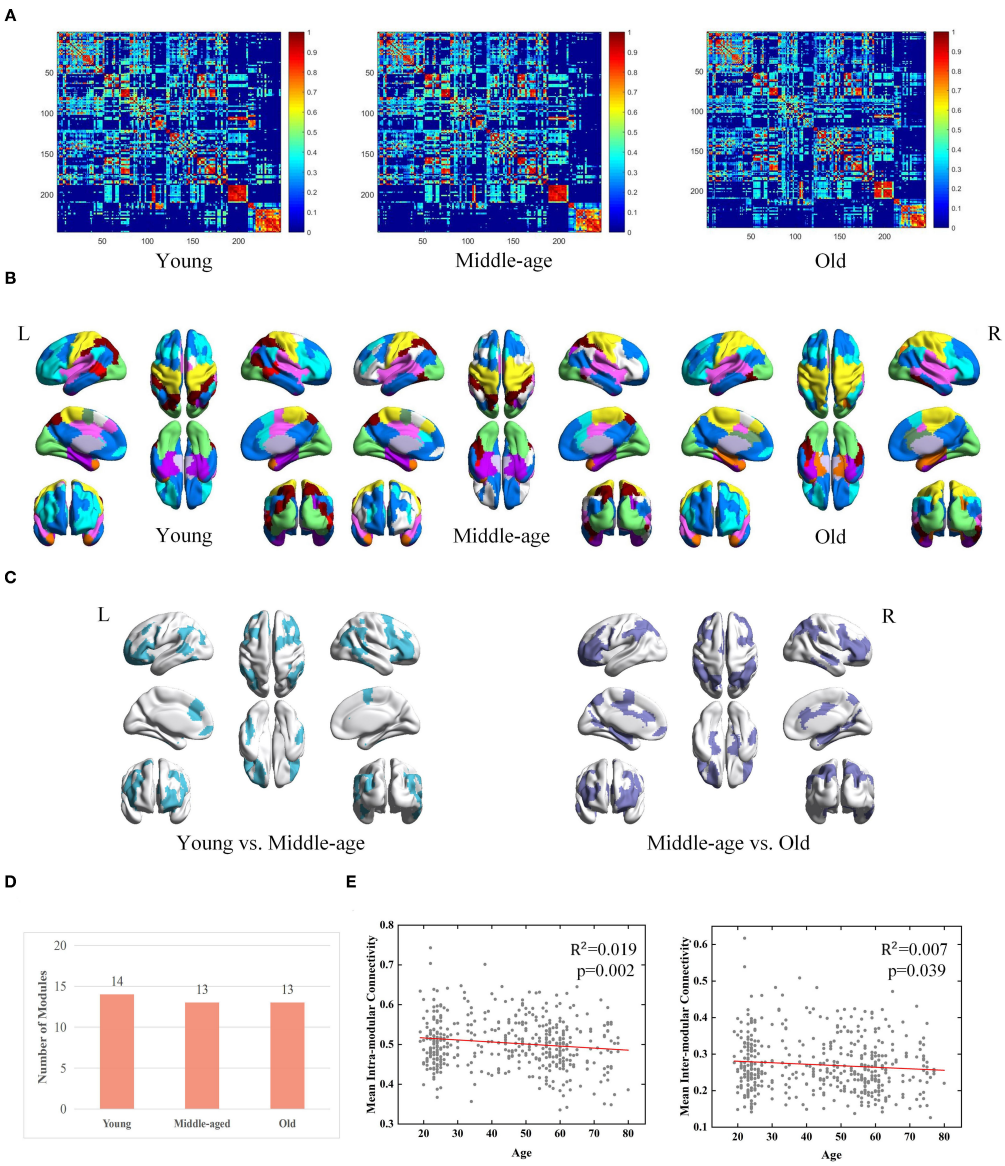
Age-associated differences in functional brain networks across three groups derived from MPP-guided method, including **(A)** group-level weighted networks, **(B)** modular structures (different colors represent different modules), **(C)** Brain regions belonging to different modules (young group vs. middle-age group and middle-age group vs. old group), **(D)** number of modules, and **(E)** age-associated trajectories of the mean intra- and inter-modular connectivities.

According to the group-level network construction method introduced in section Construction of Brain Networks, a group-level network was generated for each age group. For each group-level network, the same method was used as described in section Module Detection From Group-Level Network to obtain consistent modules. Three group-level FC networks and full views of the modular structures across three groups are visualized in Figures 3A,B. According to the detected modular structures of three groups, we find the age-associated differences of modules. In youth, the brain is separated into fourteen modules, of which six modules are also observed in the middle-age group (covering a major part of the occipital lobe, fusiform gyrus, and subcortical areas). In terms of the middle-age group and old group, seven modules are almost the same, which sit in the superior frontal gyrus, superior temporal gyrus, insular gyrus and cingulate gyrus. Brain regions belonging to different modules in the three age groups are mainly located at the frontal lobe, temporal lobe and parietal lobe (Figure 3C). It is observed that the number of modules does not differ dramatically among the three groups (Figure 3D).

Besides modular differences, we find age is negatively correlated with mean intra-modular connectivity (adjusted *R*^2^ = 0.019, *p* = 0.002) and mean inter-modular connectivity (adjusted *R*^2^ = 0.007, *p* = 0.039) (Figure 3E). Age-related effects on the inter-modular FC are observed to be weaker compared with the mean intra-modular FC.

### Module-Guided Group-Level Networks

We evaluated the necessity of the individual differences when constructing group-level network by comparing the MPP-guided group-level FC network at each group with those generated using the conventional (group-based FC average) method. Subsequently, we used the same steps as in section Module Detection From Group-Level Network to detect group-level modular structures and compared results between two methods.

**Figure 4:**
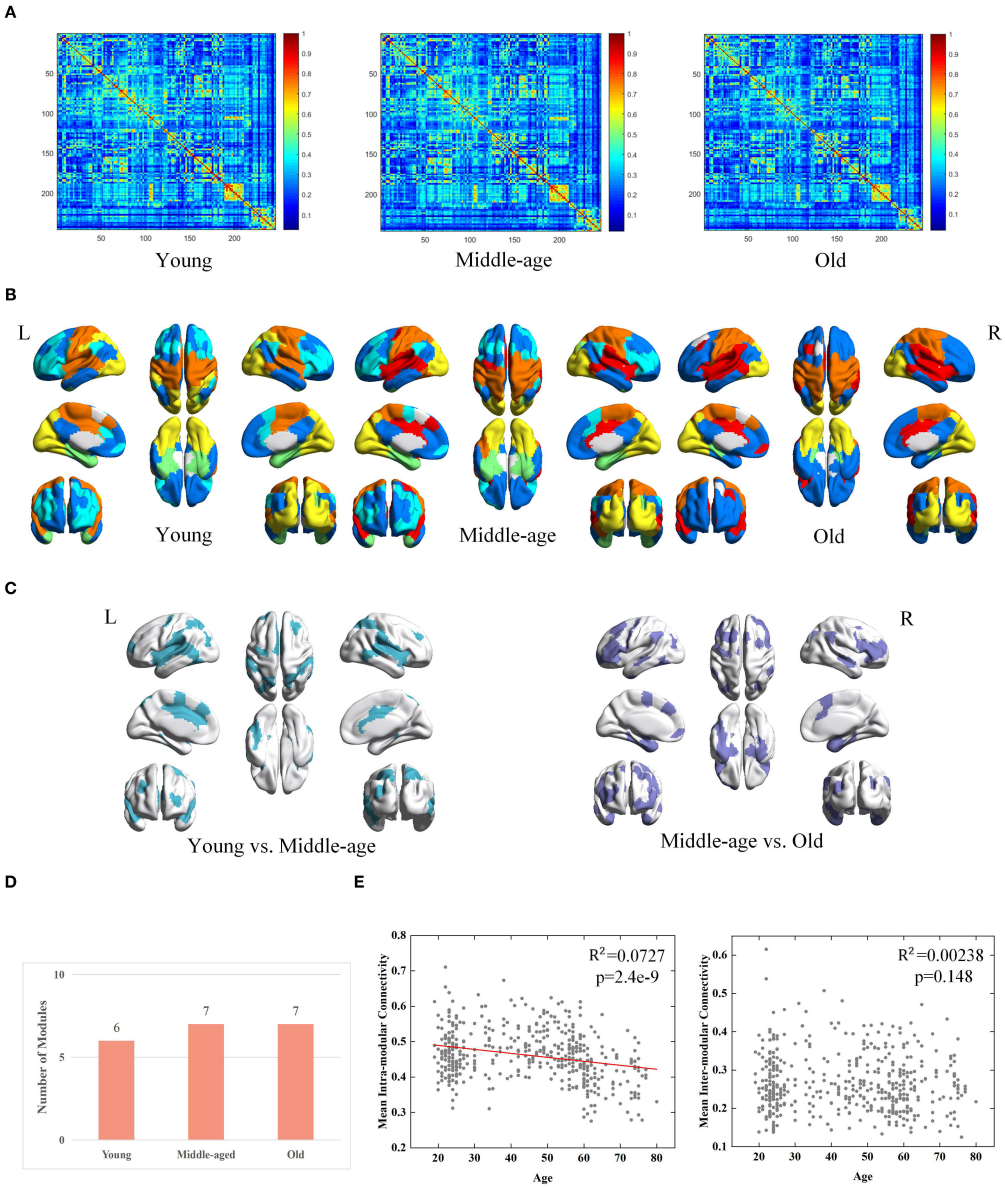
Age-associated differences in functional brain networks across three groups derived from conventional method, including **(A)** average-based group-level networks, **(B)** modular structures (different colors represent different modules), **(C)** Brain regions belonging to different modules (young group vs. middle-age group and middle-age group vs. old group), **(D)** number of modules, and **(E)** age-associated trajectories of the mean intra- and inter-modular connectivities.

Derived from conventional method, three group-level FC networks and full views of the modular structures are visualized in Figures 4A,B respectively. The group-level networks based on conventional method are observed more blurred than those from MPP-guided method. The blurred group-level networks increase the difficulty to detect modules and further lead to inconsistent modular structures at group-level. Brain regions belonging to different modules in the three age groups are mainly located at the frontal lobe, temporal lobe and parietal lobe (Figure 4C). The number of modules derived from conventional method is less than those derived from MPP-guided method, and does not differ dramatically among the three groups (Figure 4D). Based on the conventional method, we find a negative correlation between age and mean intra-modular connectivity (adjusted *R*^2^ = 0.00727, *p* = 2.4e-9), but no correlation between age and inter-modular connectivity is observed (Figure 4E). With the MPP-guided method, we obtain more consistent modules and clearer modular structure, which is more sensitive to the difference of age-related mean inter-modular connectivity than conventional method.

### Age-Associated Differences of Hubs in Brain Functional Networks

#### Differences in the Quantity and Spatial Distribution of Hubs

**Figure 5:**
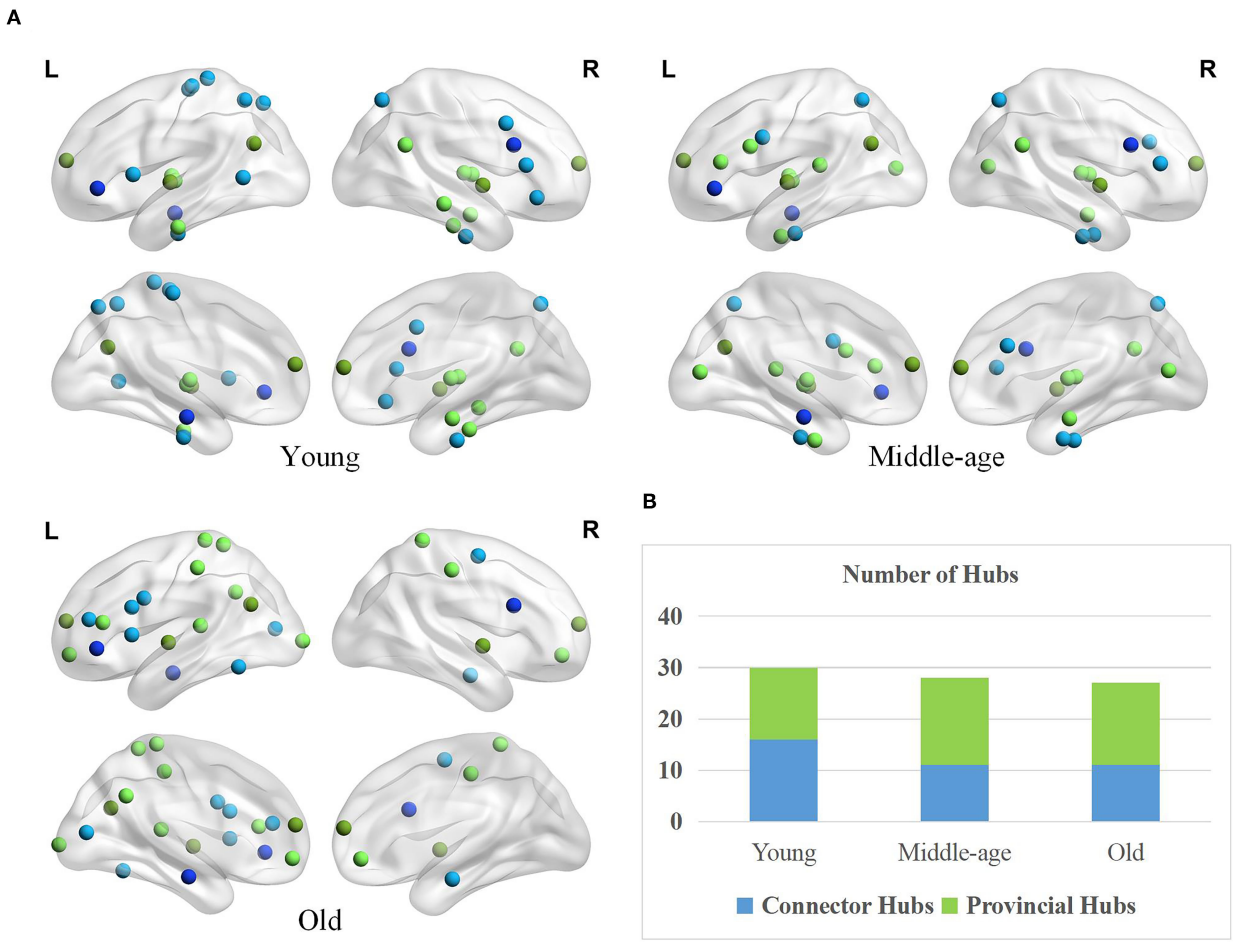
Age-associated differences in spatial distribution and the number of two types of hubs. **(A)** Spatial distributions of provincial (green) and connector hubs (blue) across three age groups. The darker color indicates the hubs remaining in the three groups and the lighter color represents the hubs that are unique to the specific group. **(B)** The number of provincial and connector hubs in three age groups.

The spatial distributions of provincial and connector hubs detected in three age groups are visualized in Figure 5A. We find the total number of two types of hubs negatively correlated with age, but the difference is not obvious (Figure 5B). Specifically, the number of connector hubs in young group is more than middle-age and old group, while the quantity of provincial hubs in middle-age group is discovered more than other two age groups. Interestingly, the ratio of hubs' quantity between the left and right hemispheres differs across three groups. The ratio of hubs' quantity of left hemisphere in the old group (i.e., 70.37%) is larger than that in young (i.e., 53.33%) and middle-age (i.e., 53.57%) group.

Besides, the spatial distribution of hubs differs greatly across three groups. Based on a priori network partition (Thomas Yeo et al., 2011), in the young age group, the hubs are mainly located in the default mode network (DMN), sensorimotor network (SN), and dorsal attention network (DAN). Different from young age group, the hubs in middle-age group are widely distributed in many subnetworks. In old age, hubs mainly concentrated on the frontoparietal network and some higher-order cognitive networks (Chong et al., 2019). Differernt from young group, the provincial hubs in middle-age group newly appear in the inferior frontal gyrus (IFG) and medioventral occipital cortex (MVOcC) while the connector hubs disappear in some subregions such as the orbital gyrus (OrG), paracentral lobule (PrG), and middle temporal gyrus (MTG). Compared with the middle-age group, the provincial hubs in old age disappear in the medioventral occipital cortex (MVOcC) and some subcortical areas principally while newly appear in the superior parietal lobule (SPL), orbital gyrus (OrG), and postcentral gyrus (PoG). In addition, the connector hubs in old age mainly disappear in the fusiform gyrus (FuG) and superior parietal lobule (SPL) and appear in the inferior temporal gyrus (ITG) as well as middle frontal gyrus (MFG), in comparison to middle-age group.

#### Hubs' Age-Associated Trajectories of WD and PC

In addition to the location and quantity differences of hubs, the age-associated trajectories of WD and PC were further calculated for each hub to quantify their roles in the modular topology. The relationship between each of the two attributes and age may represent some kind of conversion of hubs across three age groups. For instance, the ROIs whose WD negatively correlated with age are probably to convert from hubs to non-hubs since hubs usually have high WD. On the contrary, if WD is positively related to age, the ROIs might convert from non-hubs to hubs. The ROIs with PC that are significantly positively correlated with age possibly convert from provincial hubs to connector hubs for the reason that connector hubs usually have high WD as well as PC while the ROIs' unchanged high WD may indicate that these regions were provincial hubs before. For the ROIs whose WD and PC both have no correlation with age, they are likely to maintain the state of provincial hubs or connector hubs (Wen et al., 2019).

**Figure 6:**
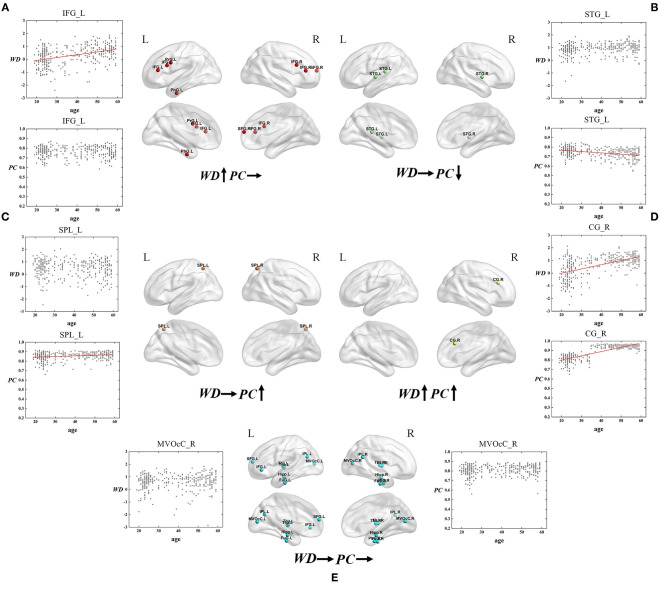
The spatial distributions of hubs at Young - Middle age stage with **(A)** only WD has a positive correlation with age; **(B)** only PC has a negative correlation with age; **(C)** only PC has a positive correlation with age; **(D)** Both WD and PC have a positive correlation with age; **(E)** Both WD and PC have no correlation with age. Red lines fit the curve with statistically significant correlation.

**Figure 7:**
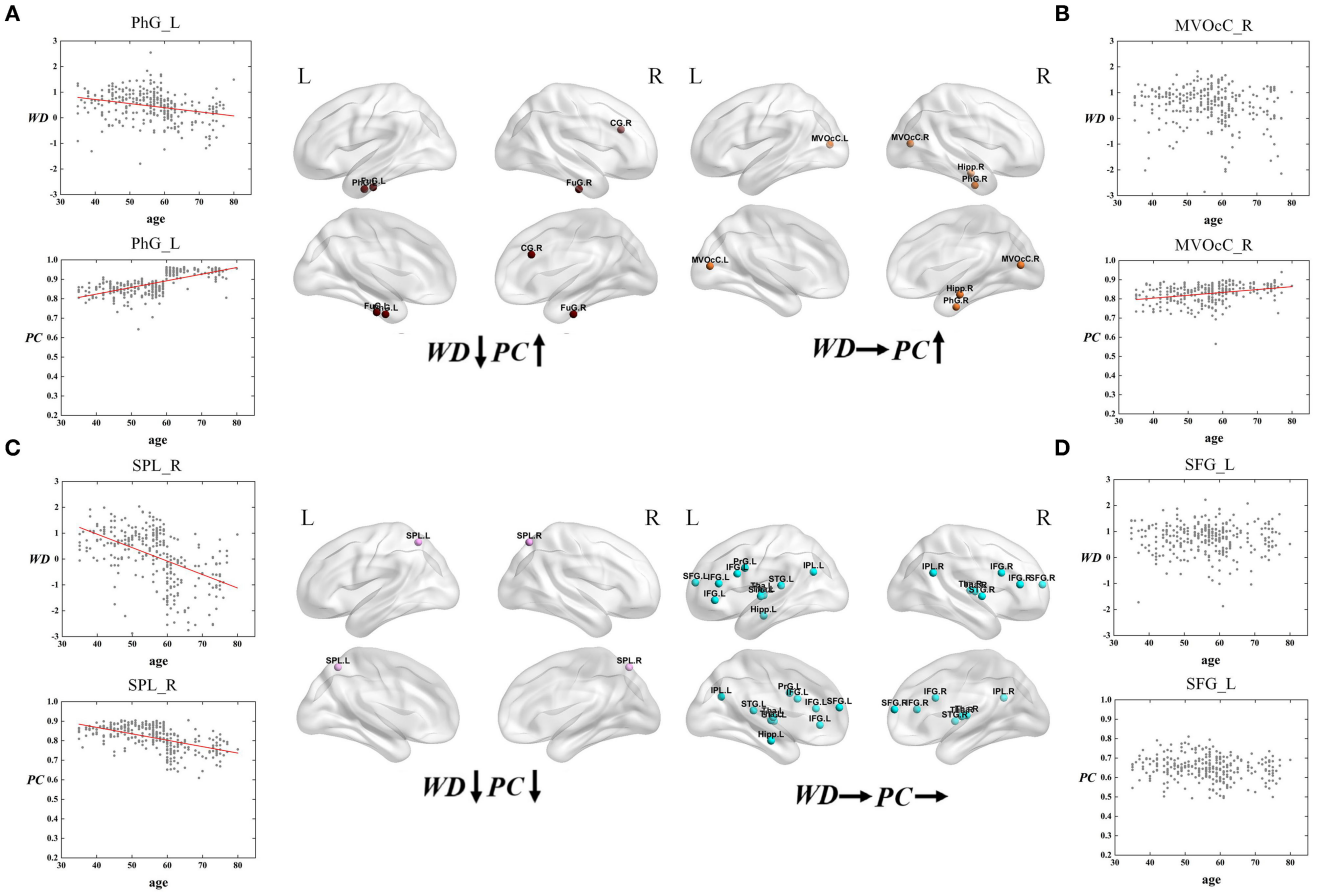
The spatial distributions of hubs at Middle - Old age stage with **(A)** WD has a negative correlation with age but PC has a positive correlation with age; **(B)** only PC has a positive correlation with age; **(C)** both WD and PC have a negative correlation with age; **(D)** both WD and PC have no correlation with age. Red lines fit the curve with statistically significant correaltion.

Based on the thoughts above, taking the hubs in the middle-age group as the ROI, the three groups of young, middle-age, and old were divided into Young - Middle age stage and Middle - Old age stage for comparison and discussion. We classified the ROIs into five categories combined with the relationship between age and WD/PC at Young - Middle age stage (Figure 6, Table 1) and divided the ROIs into four categories at the Middle - Old age stage (Figure 7, Table 2). For each category, one brain region was selected and the age-associated trajectories of its WD and PC are plotted.

**Table 1:** Fitted correlation curves of WD and PC of selected hubs in the Young - Middle age stage.

**Brain regions**	**WD**				**PC**			
	**Model**	**Intercept**	**Slope**	***p*(slope)**	**Model**	**Intercept**	**Slope**	***p*(slope)**
**A. Only WD has a positive correlation with age**								
SFG_R	**Linear**	0.3166	1.14E-02	1.02E-06	Linear	0.6587	−7.83E-05	7.56E-01
IFG_L	**Linear**	−0.4467	1.76E-02	6.25E-10	Linear	0.7703	7.93E-05	6.86E-01
IFG_R	**Linear**	−0.2300	1.67E-02	7.92E-09	Linear	0.7759	3.15E-04	8.97E-02
IFG_L	**Linear**	−0.5172	2.18E-02	1.05E-15	Linear	0.7699	−1.27E-04	5.38E-01
IFG_R	**Linear**	−0.3084	1.74E-02	1.27E-08	Linear	0.7736	1.92E-04	3.61E-01
PrG_L	**Linear**	−0.2378	1.94E-02	2.35E-10	Linear	0.7967	−1.66E-05	9.28E-01
PhG_L	**Linear**	0.1554	9.55E-03	4.18E-05	Linear	0.8398	1.23E-04	4.19E-01
**B. Only PC has a negative correlation with age**								
STG_L	Linear	0.6791	6.01E-03	4.73E-04	**Linear**	0.7948	−1.39E-03	1.45E-12
STG_L	Linear	1.3303	−1.17E-03	3.71E-01	**Linear**	0.7805	−1.24E-03	4.93E-10
STG_R	Linear	1.4427	−3.79E-03	3.59E-03	**Linear**	0.7793	−1.41E-03	1.24E-11
**C. Only PC has a positive correlation with age**								
SPL_R	Linear	0.8997	−5.08E-03	4.92E-02	**Linear**	0.8069	8.06E-04	2.08E-06
SPL_L	Linear	0.5717	−2.38E-03	3.73E-01	**Linear**	0.8302	6.44E-04	7.60E-05
**D. Both WD and PC have a positive correlation with age**								
CG_R	**Linear**	−0.67885	1.34E-01	2.40E-33	**Linear**	0.7095	0.0044733	6.53E-90
**E. Both WD and PC have no correlation with age**								
SFG_L	Linear	0.5461	4.03E-03	4.56E-02	Linear	0.6714	−2.65E-04	2.60E-01
IFG_L	Linear	0.2758	4.86E-03	1.02E-01	Linear	0.7639	1.59E-04	4.47E-01
FuG_L	Linear	1.2242	1.55E-04	9.18E-01	Linear	0.8453	6.40E-05	6.23E-01
FuG_R	Linear	1.0124	1.40E-03	4.76E-01	Linear	0.8442	1.98E-04	1.39E-01
PhG_R	Linear	0.1473	7.54E-03	2.50E-03	Linear	0.8339	3.42E-04	2.11E-02
IPL_L	Linear	1.2856	−3.94E-03	3.92E-02	Linear	0.6167	4.08E-04	1.43E-01
IPL_R	Linear	0.8896	−5.23E-03	5.91E-02	Linear	0.6387	5.12E-04	9.23E-02
MVOcC _L	Linear	0.5751	5.38E-03	1.45E-02	Linear	0.7770	5.82E-04	7.40E-03
MVOcC _R	Linear	0.5002	3.74E-03	1.10E-01	Linear	0.7896	4.93E-04	1.52E-02
Hipp_L	Linear	0.8700	1.67E-03	4.89E-01	Linear	0.8420	−1.81E-04	2.81E-01
Hipp_R	Linear	0.9566	−3.95E-03	9.86E-02	Linear	0.8250	1.96E-05	9.18E-01
Tha_L	Linear	1.1228	7.78E-05	9.51E-01	Linear	0.7755	−1.08E-04	7.82E-01
Tha_R	Linear	1.1637	−1.56E-03	2.86E-01	Linear	0.7791	−3.53E-04	3.74E-01
Tha_L	Linear	1.0651	9.72E-04	3.38E-01	Linear	0.7935	9.30E-05	7.76E-01
Tha_R	Linear	1.2163	−1.25E-03	3.02E-01	Linear	0.7884	−2.03E-04	5.86E-01

*The same brain region names in the table represent the different parts of the corresponding brain areas. The statistically significant correlation between WD/PC and age is in bold*.

**Table 2:** Fitted correlation curves of WD and PC of selected hubs in the Middle - Old age stage.

**Brain regions**	**WD**				**PC**			
	**Model**	**Intercept**	**Slope**	**p(slope)**	**Model**	**Intercept**	**Slope**	**p(slope)**
**A. WD has a negative correlation with age but PC has a positive correlation with age**								
FuG_L	**Linear**	2.033	−0.018016	2.48E-11	**Linear**	0.6845	3.52E-03	7.35E-39
FuG_R	**Linear**	1.5661	−0.010957	7.47E-04	**Linear**	0.71058	0.0031	1.23E-35
PhG_L	**Linear**	1.3608	−1.61E-02	1.27E-06	**Linear**	0.6871	3.42E-03	1.61E-34
CG_R	**Linear**	1.9304	−1.96E-02	7.43E-08	**Linear**	0.89808	0.0010	2.59E-22
**B. Only PC has a positive correlation with age**								
PhG_R	Linear	0.7218	−4.91E-03	1.82E-01	**Linear**	0.6973	3.30E-03	4.00E-37
MVOcC _L	Linear	0.9248	−2.86E-03	4.52E-01	**Linear**	0.7396	1.43E-03	5.33E-07
MVOcC _R	Linear	1.0980	−1.05E-02	1.55E-02	**Linear**	0.7442	1.49E-03	1.75E-08
Hipp_R	Linear	1.0498	−4.01E-03	3.00E-01	**Linear**	0.7498	1.62E-03	8.70E-08
**C. Both WD and PC have a negative correlation with age**								
SPL_R	**Linear**	3.0372	−5.19E-02	2.03E-20	**Linear**	0.99986	−0.0033	2.47E-23
SPL_L	**Linear**	1.2422	−1.71E-02	8.77E-05	**Linear**	1.0797	−0.0047	3.23E-32
**D. Both WD and PC have no correlation with age**								
SFG_L	Linear	0.7755	−1.03E-03	7.53E-01	Linear	0.6996	−9.02E-04	1.01E-02
SFG_R	Linear	1.1316	−4.45E-03	1.68E-01	Linear	0.7164	−1.31E-03	5.34E-04
IFG_L	Linear	0.2835	3.64E-03	3.47E-01	Linear	0.7734	−4.50E-05	8.86E-01
IFG_R	Linear	0.9621	−6.15E-03	9.09E-02	Linear	0.7982	−1.07E-04	7.15E-01
IFG_L	Linear	0.3834	3.67E-03	2.99E-01	Linear	0.7678	−8.34E-05	8.07E-01
IFG_R	Linear	0.7930	−4.24E-03	3.33E-01	Linear	0.7721	1.92E-04	5.66E-01
IFG_L	Linear	0.5344	−4.24E-04	9.15E-01	Linear	0.7859	−2.86E-04	4.03E-01
PrG_L	Linear	0.3053	8.73E-03	3.14E-02	Linear	0.8197	−4.91E-04	9.57E-02
STG_L	Linear	1.0985	−2.18E-03	3.59E-01	Linear	0.7328	−1.32E-04	6.79E-01
STG_L	Linear	1.5950	−6.78E-03	2.32E-03	Linear	0.7513	−6.85E-04	4.35E-02
STG_R	Linear	1.5164	−5.33E-03	1.17E-02	Linear	0.7401	−6.20E-04	8.18E-02
IPL_L	Linear	1.4648	−7.55E-03	1.34E-02	Linear	0.6605	−5.66E-04	1.34E-01
IPL_R	Linear	1.1304	−9.21E-03	2.11E-02	Linear	0.6862	−5.40E-04	2.02E-01
Hipp_L	Linear	0.6599	6.72E-03	7.23E-02	Linear	0.7898	9.93E-04	9.66E-04
Tha_L	Linear	1.3147	−4.08E-03	7.34E-02	Linear	0.7124	1.11E-03	5.08E-02
Tha_R	Linear	1.2233	−2.95E-03	2.34E-01	Linear	0.7151	8.92E-04	1.16E-01
Tha_L	Linear	0.9598	3.08E-03	5.93E-02	Linear	0.7489	9.55E-04	3.44E-02
Tha_R	Linear	1.2701	−2.51E-03	2.15E-01	Linear	0.7442	6.42E-04	2.39E-01

*The same brain region names in the table represent the different parts of the corresponding brain areas. The statistically significant correlation between WD/PC and age is in bold*.

As for the Young - Middle age stage, of all 28 ROIs, 25% only have a positive correlation between age and WD (the first category), which indicates these regions play an increasingly important role in their within-modular connections. Compared with young, most of the regions in middle-age group convert from non-hubs to hubs, which are mainly located at inferior frontal gyrus and right superior frontal gyrus (Figure 6A). As shown in Figure 6B, three regions of 28 ROIs only have a negative correlation between age and PC (the second category), which play an increasingly weak role in inter-modular connections. Most of these regions are provincial hubs in both young and middle-age groups, which sit in superior temporal gyrus. The third category consists of two ROIs, whose PC are positively correlated with age. Similar to the second category, the two regions are connector hubs in both young and middle-age groups which exhibit an increasingly important role in inter-modular connections with the location in superior parietal lobule (Figure 6C). Figure 6D shows one region whose WD and PC both have a positive correlation with age (the fourth category). The region converts from non-hub to connector hub and has an increasingly effect on both within-modular and inter-modular connections, which sits in the right cingulate gyrus. The last category includes most regions (53.6%) whose WD and PC both have no correlation with age. The role of these hubs in young is almost the same as in middle-age (provincial hubs or connector hubs). These regions are widely distributed, which are mainly located at the inferior parietal lobule, medioventral occipital cortex, fusiform gyrus as well as thalamus and hippocampus (Figure 6E).

Similarly, the age-associated trajectories of WD and PC in the Middle - Old age stage are studied. Four ROIs have a negative correlation between age and WD as well as a positive correlation between age and PC, which indicates these regions play an increasingly important role in their inter-modular connections as well as a weaker role in within-modular connections. All of these ROIs convert from hubs to non-hubs, which are located at the temporal lobe and right cingulate gyrus (Figure 7A). The second category consists of four ROIs, whose PC are positively correlated with age. Some of these regions are already detected as provincial hubs in middle-age because of their high WD. With the positive correlation between age and PC, many of them tend to convert from provincial hubs to connector hubs in the old group, which are mainly located at the medioventral occipital cortex, right parahippocampal gyrus as well as right hippocampus (Figure 7B). As shown in Figure 7C, two regions' WD and PC are inversely associated with age (the third category). These two regions are identified as connector hubs in middle-age because of their high WD and PC. The negative correlation between age and WD/PC results in the conversion to non-hubs in old age group. The last category includes most regions (64.3%) whose WD and PC both have no correlation with age. The role of these hubs in middle-age is almost the same as in old, which means they are playing an important role at this stage in brain functional networks. These regions have wide distribution, which are mainly located at the inferior frontal gyrus, superior frontal gyrus, inferior parietal lobule, and thalamus (Figure 7D).

## Discussion

In the present study, we combine the group-level FC networks and two crucial network topological measures (modules and hubs) to investigate the age-associated differences of brain functional networks using a large cross-sectional lifespan dataset. Furthermore, the ROI-wise analysis of WD and PC characterized different age-associated trajectories of ROIs as well as hub conversion.

### Weakened Functional Segregation and Integration

The human brain is functionally organized into a complex network to facilitate the effective segregation and integration of information processing (Sporns et al., 2005; Zhu et al., 2018). Previous works have indicated that each module of the functional network is associated with specific cognitive/behavior function (Bertolero et al., 2015). Brain is parsed into coherent sub-systems by functional modules, which allows for both functional integration and segregation among different brain areas (Puxeddu et al., 2020; Zheng et al., 2020). To quantify the functional segregation and integration, the analysis of detected modular structures and hubs was performed (Van den Heuvel and Sporns, 2013b; Bertolero et al., 2015; Cohen and D'Esposito, 2016). We find the intra- and inter-modular connectivities negatively correlated with age, which suggests weakened functional segregation and integration. The weakened network segregation in this study is consistent with recent research showing attenuated local efficiency with age, which suggests that aging is relevant to the reduced ability of local information processing and specialized functions (Cao et al., 2014; Geerligs et al., 2015). For network integration, many research similarly demonstrated lower functional integration with age (Chong et al., 2019; Oschmann and Gawryluk, 2020). Additionally, age-related effects on integration were weaker than on segregation in this study, which is in coherence with (Chong et al., 2019). We find some support for age-related decrease in functional segregation/ integration and reveal that the age-associated differences of modular organization may be driven by weakened network segregation and integration.

Brain function is often considered as a balance between segregate and integrative information processing (Bassett and Gazzaniga, 2011; Bertolero et al., 2015; Deco et al., 2015). Based on this theory, each module has a strong ability of local information processing and specialized functions. As different modules are responsible for relatively specialized functions, connector hubs are thought to carry out efficiency information exchange between modules whereas provincial hubs integrate information within modules to support the specialized function (Bertolero et al., 2015). The smaller quantity of connector hubs in middle-age and old group provides additional evidence for the decreasing functional integration because these regions are mostly responsible for information integration among different modules. Meanwhile, it probably results in the reduced network information processing efficiency of the aging process in brain functional network. The altered number of provincial hubs in three groups is possibly related to the specialization of information processing that presents an inverted U-shaped trajectory from young to old (Cao et al., 2014). The larger quantity of provincial hubs in middle-age and old group seems to reflect a compensation for smaller quantity of connector hubs to maintain performance of information processing (Kurth et al., 2016), leading to little difference in total number of hubs across three groups. Additionally, the old group showed greater ratio of hubs' quantity of left hemisphere than young and middle-age group, suggesting that the right hemisphere exhibits greater age-related reduction compared with the left hemisphere (Coppi et al., 2014; Lebedeva et al., 2017), which provides support for the right hemi-aging model (Dolcos et al., 2002).

### Different Age-Associated Trajectories for Provincial and Connector Hubs

In the present study, we detected two types of hubs (provincial and connector hubs) according to the group-level modular structure of the middle-age group and investigated the age-associated trajectories of WD and PC of these hubs in two stages (Young - Middle and Middle - Old age stage).

#### Different Age-Associated Trajectories in Young - Middle Age Stage

In terms of Young - Middle age stage, combining the first and fourth categories (both have a positive correaltion between age and WD), we found the relevant brain regions are mainly distributed in the right superior frontal gyrus, inferior frontal gyrus, and right cingulate gyrus (Figures 6A,D). These regions mainly relate to the dorsal attention network (Corbetta et al., 2002) and frontoparietal network (Vincent et al., 2008), both supporting sustained attention and working memory (Brissenden et al., 2016). Most of these regions convert from non-hubs to hubs with an increasing role in their within-modular connections. As high WD results in high co-activations of nodes in the same module (Messé et al., 2018), the positive correaltion between age and WD indicates more activation in these regions in middle-age, which may be the compensation for the participation of other weak circuits (Reuter-Lorenz and Park, 2014). The third and fourth categories (both have a positive correaltion between age and PC) are located in higher-order cognitive networks, which show an increasingly important role in inter-modular connections (Figures 6C,D). The positive correaltion between age and PC in these regions may cause enhanced higher-order cognitive functions in middle-age since PC has been proved to have a positive correlation with cognitive function (de Haan et al., 2012). Only the second category shows PC negatively correlated with age and the involved brain regions are distributed in the superior temporal gyrus, part of the somatomotor network (Figure 6B). Though most of these regions are provincial hubs in the young and middle-age group, the negative correaltion between age and PC may lead to the loss of action recognition, episodic memory, and spatial navigation in middle-age (Li et al., 2015). Such findings manifest that some intra-modular connections tend to enhance in the middle-age group compared with young, but the consistent trend in inter-modular connections is not found.

#### Different Age-Associated Trajectories in Middle – Old Age Stage

For the Middle – Old age stage, the first and second categories show a positive correaltion between age and PC, which are mainly located at the temporal lobe, right cingulate gyrus, medioventral occipital cortex, and right parahippocampal gyrus (Figures 7A,B). These brain regions principally correspond to the limbic and visual networks. The positive correaltion between age and PC in these regions indicates the enhanced inter-modular connections in old age. As a result, some of these regions convert from provincial hubs to connector hubs. The first category whose WD has a negative correlation with age but PC has a positive correlation with age of the limbic network may represent maintaining a balance between the local specialization and global integration of the information process in old age. Combining the first and third type (both have a negative correaltion between age and WD), the relevant regions were observed mainly distributed in the temporal lobe, right cingulate gyrus, and superior parietal lobule (Figures 7A,C), which were related to the limbic network and dorsal attention network. Most of these regions change from hubs to non-hubs with reduced within-modular connections, which may be caused by the low co-activations of nodes within the module in old age (Messé et al., 2018). These hubs require further study because of the loss of essential roles in the network topology. Of note, the third category shows two regions whose WD and PC both have a negative correlation with age. They are located in the dorsal attention network and both convert from connector hubs to non-hubs (Figure 7C). The negative correlation between age and PC in these regions may lead to weaked sustained attention and working memory in old age (Brissenden et al., 2016). The findings suggest the topological roles of these hubs have converted greatly between middle-age and old group, which will help to understand the mechanism associated with the aging brain. Our findings illustrate that intra-modular connections tend to decline in the old group compared with middle-age, but some inter-modular connections could have protracted development during this period. Additionally, functional segregation becomes weak during this period while some inter-modular connections keep developing to balance the modular structure in the brain network (Chen et al., 2011).

## Conclusion and Limitations

In this study, we applied a module-guided group-level network construction method to combine individual-level networks to form a weighted group-level network for each group. Based on the analysis of group-level network modular structure, the intra- and inter-modular connectivities were observed negatively correlated with age, manifesting the weakened functional segregation and integration. Meanwhile, the negative correlation between age and intra-/inter-modular connectivity suggested less efficient information communication of brain networks with age. Furthermore, our findings showed detailed age-associated trajectories of hubs in functional brain networks. In sum, this study investigates the age-associated differences of modular structure and hubs in functional networks across three age groups and provides a new perspective and useful information to better understand the normal aging of the brain.

Nevertheless, our study also had some limitations. First, there are no psychophysiology tests included in the database that physiological states or neuropsychological performance are scored. Such information facilitates a more meaningful understanding of the relationship between age and cognition. Second, due to the limited sample size and large age range, the participants in our study were divided into three age groups and our results are not detailed enough across 60 years. Besides, the difference of the hubs' quantity was obtained based on group-level network in our study, future research should analyze the statistical significance of the difference in age-related hubs' quantity. Furthermore, due to the limitations of the database, we analyzed hubs/modules based on group-level networks even though the participants don't match up across groups. Future research should apply the research to longitudinal data for more accurate result.

## Data Availability Statement

Publicly available datasets were analyzed in this study. This data can be found here: The dataset used in this study is accessible from International Data-sharing Initiative (http://fcon_1000.projects.nitrc.org/indi/retro/sald.html).

## Ethics Statement

The studies involving human participants were reviewed and approved by the Research Ethics Committee of the Brain Imaging Center of Southwest University. The participants provided their written informed consent to participate in this study.

## Author Contributions

All authors listed have made a substantial, direct and intellectual contribution to the work, and approved it for publication.

## Conflict of Interest

The authors declare that the research was conducted in the absence of any commercial or financial relationships that could be construed as a potential conflict of interest.
