# Supplementary material for: Age-Associated Differences of Modules and Hubs in Brain Functional Networks
Source: Front Aging Neurosci. 2021 Jan 18;12:607445. doi: 10.3389/fnagi.2020.607445 (PMC7848126; doi:10.3389/fnagi.2020.607445)
Supplement: Supplementary file 1 [file Table_1.DOCX]

**Threshold selection**

The threshold $\mathrm{thr}_{i}$ used to sparsify the individual-level networks may have influence on individual-level module detection and the construction of group-level FC networks. To test such influences, we changed $\mathrm{thr}_{i}$ from 0.05 to 0.15 with 0.01 as an increment. At each setting, we detected the individual-level modular structures and further constructed group-level FC networks for three groups, as introduced in Section 2.4 and 2.5. Subsequently, we detected the corresponding group-level modular structure and calculated its similarity to the result with $\mathrm{thr}_{i}$ = 0.1 by using NMI in each group, respectively (see Section 2.7). All NMI values were greater than 0.8, indicating high similarities of modular structures between different settings of $\mathrm{thr}_{i}$ (Wen et al., 2019) (**Figure** **S1 A**). Such results were also supported by similar modularity (Q) values of the modular structures with different $\mathrm{thr}_{i}$ in each group (**Figure** **S1 B**).


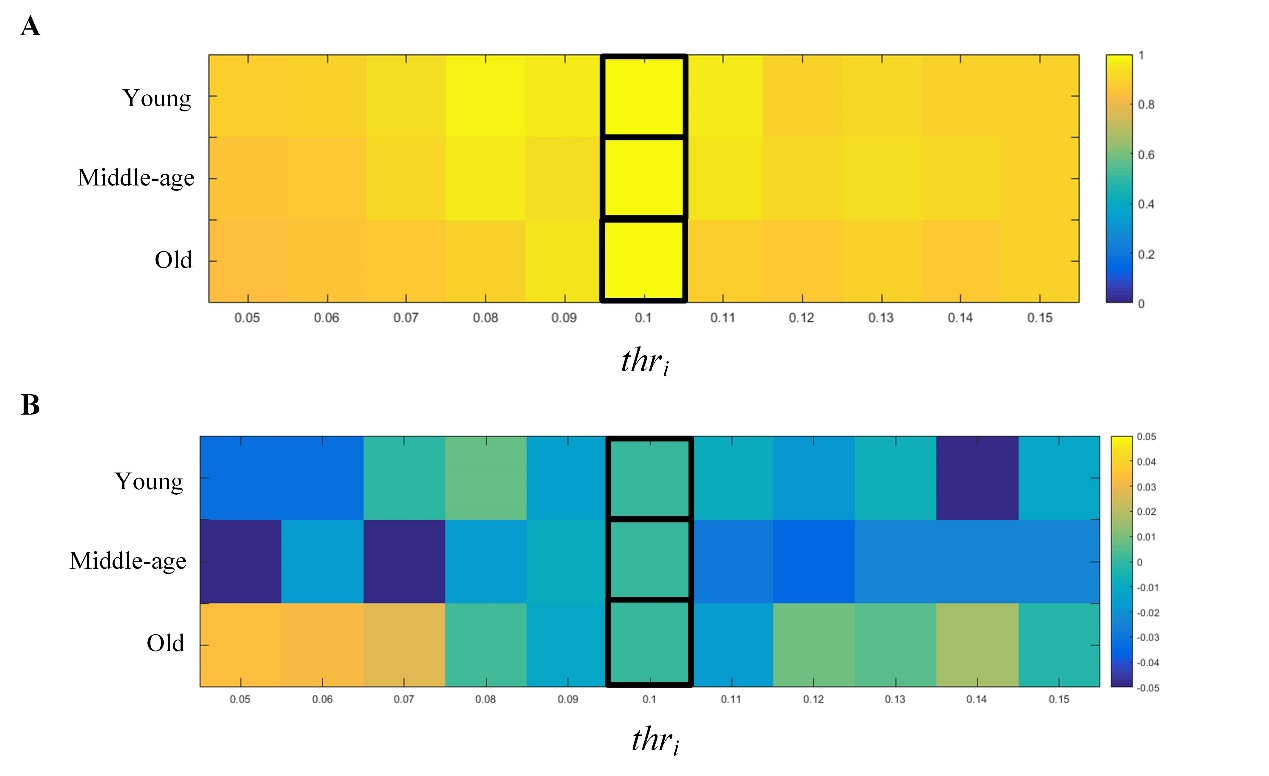


**FIGURE S1 |** Effects of $\mathrm{thr}_{i}$ on the group-level networks. We set $\mathrm{thr}_{i}$ as [0.05, 0.06, 0.07, 0.08, 0.09, 0.10, 0.11, 0.12, 0.13, 0.14, 0.15] for individual-level modular detection and further constructed the group-level FC network for each group. We then evaluate the difference between the networks adopted in experiment parts (i.e., $\mathrm{thr}_{i}$ is set to 0.10) and the ones with other settings of $\mathrm{thr}_{i}$ at each age group by measuring similarities of the detected modular structures. **(A)** NMI values of the corresponding group-level modular structure from different $\mathrm{thr}_{i}$ compared with $\mathrm{thr}_{i}$ = 0.1; **(B)** Q values of the corresponding group-level modular structure from different $\mathrm{thr}_{i}$ compared with $\mathrm{thr}_{i}$ = 0.1. The referred network is labelled with black squares.

To test the $\mathrm{thr}_{l}$ and $\mathrm{thr}_{h}$ for group-level MPP matrices, we respectively set the $\mathrm{thr}_{l}$from 0.05 to 0.25 and $\mathrm{thr}_{h}$from 0.45 to 0.65 with a step of 0.05. For different combinations, after re-generating group-level networks for three age groups, the detected consistent modular structures were compared with that constructed with $\mathrm{thr}_{l}$ = 0.1 and $\mathrm{thr}_{h}$ = 0.5 using NMI and Q in each group. All NMI values were almost greater than 0.8, indicating high similarities of modular structures between different settings of $\mathrm{thr}_{i}$ (**Figure** **S2 A**). Such results were also supported by similar modularity (Q) values of the modular structures with different $\mathrm{thr}_{i}$ in each group (**Figure** **S2 B**).


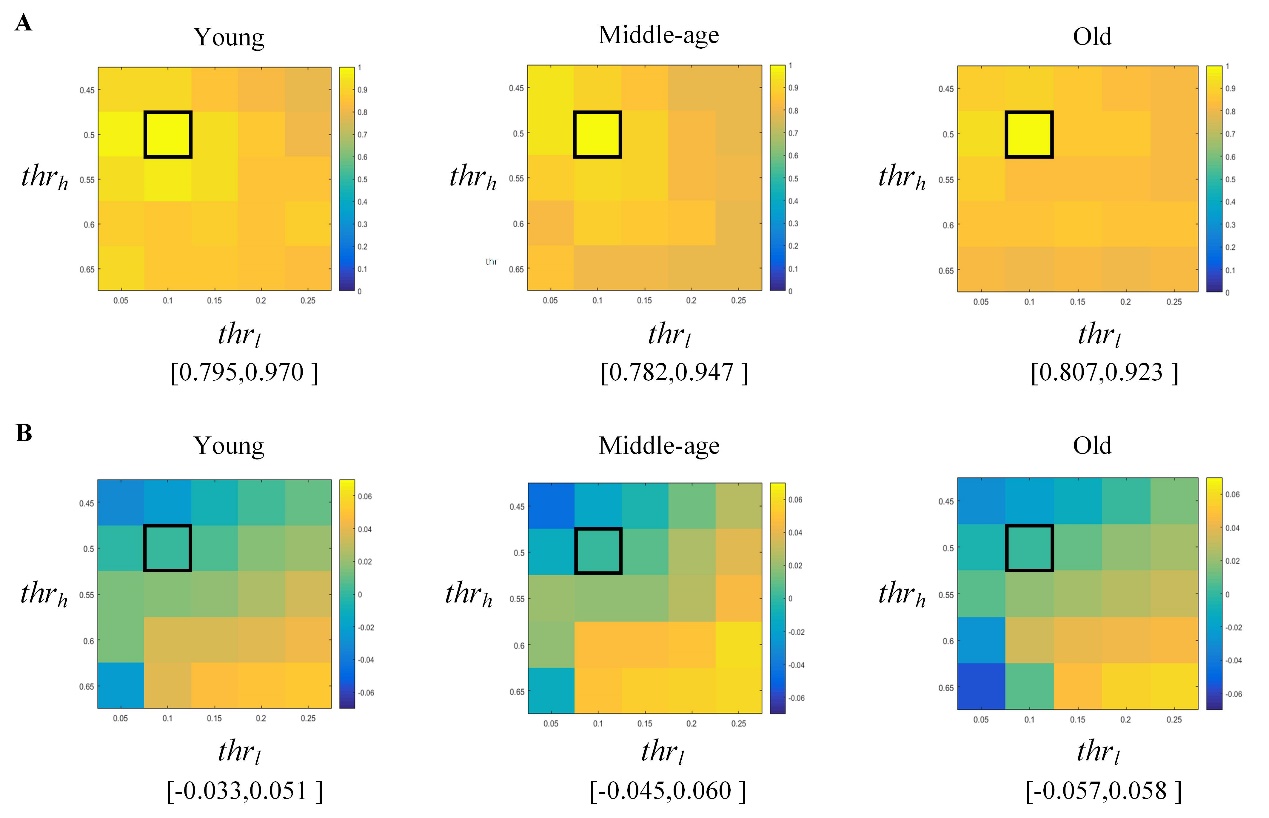


**FIGURE S2 |** Effects of $\mathrm{thr}_{l}$and $\mathrm{thr}_{h}$ on the group-level networks. We set $\mathrm{thr}_{l}$and $\mathrm{thr}_{h}$ as [0.05, 0.10, 0.15, 0.20, 0.25] and [0.45, 0.50, 0.55, 0.60, 0.65] respectively and generate a set of networks based on different combinations of these two parameters. We then evaluate the difference between the networks adopted in experiment parts (i.e., $\mathrm{thr}_{l}$and $\mathrm{thr}_{h}$ are set to 0.10 and 0.50) and the ones with other settings of $\mathrm{thr}_{l}$and $\mathrm{thr}_{h}$ at each age group by measuring similarities of the detected modular structures. **(A)** NMI values of the corresponding group-level modular structure from different $\mathrm{thr}_{l}$and $\mathrm{thr}_{h}$ compared with $\mathrm{thr}_{l}$ = 0.1 and $\mathrm{thr}_{h}$ = 0.5; **(B)** Q values of the corresponding group-level modular structure from different $\mathrm{thr}_{l}$and $\mathrm{thr}_{h}$ compared with $\mathrm{thr}_{l}$ = 0.1 and $\mathrm{thr}_{h}$ = 0.5. The referred network is labelled with black squares.

To test the $\mathrm{thr}_{WD}$ and $\mathrm{thr}_{PC}$ for hub classification. By fixing $\mathrm{thr}_{PC}$ to 0.55 and varying $\mathrm{thr}_{WD}$ from 0.8 to 1.1 with a step of 0.1, the hubs in each group did not change a lot in the spatial distribution or quantity and $\mathrm{thr}_{WD}$ settings of hubs showed similar differences (**Figure** **S3 A**). Similarly, by fixing $\mathrm{thr}_{WD}$ to 1.0 and increasing $\mathrm{thr}_{PC}$ from 0.5 to 0.65 with 0.05 as an increment, the impacts of $\mathrm{thr}_{PC}$ on the hub assessment were also tested. Although the number of connector hubs decreased as $\mathrm{thr}_{PC}$ increased, the setting of $\mathrm{thr}_{PC}$ has no effect on the difference in two types of hubs’ quantity between three age groups (**Figure** **S3 B**).


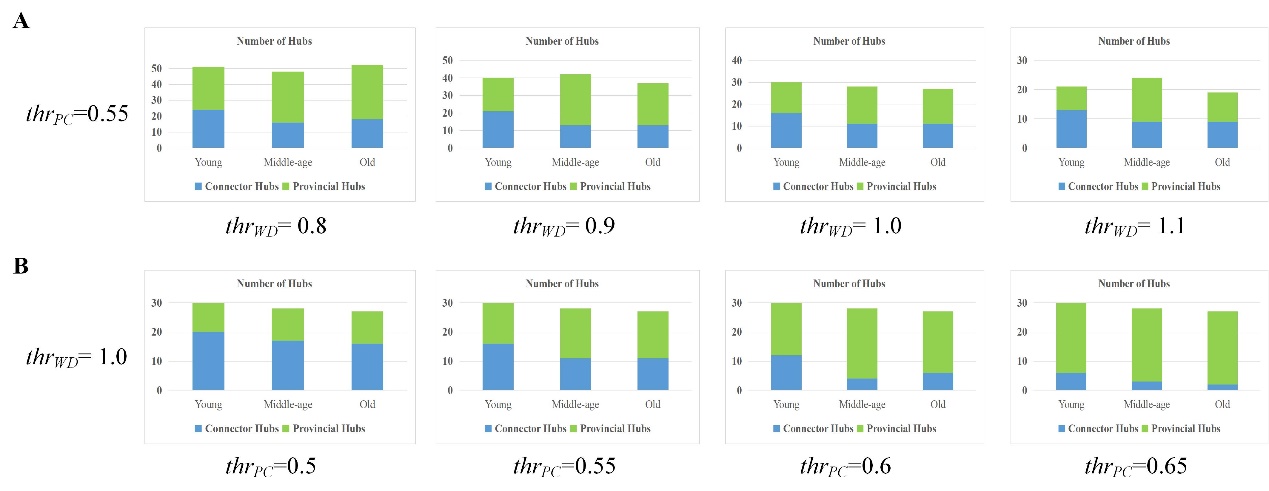


**FIGURE S3 |** Effects of $\mathrm{thr}_{WD}$ and $\mathrm{thr}_{PC}$ on the hub classification. **(A)** The impacts of $\mathrm{thr}_{WD}$ on the hub assessment by fixing $\mathrm{thr}_{PC}$ to 0.55 and varying $\mathrm{thr}_{WD}$ from 0.8 to 1.1 with a step of 0.1. **(B)** The impacts of $\mathrm{thr}_{PC}$ on the hub assessment by fixing $\mathrm{thr}_{WD}$ to 1.0 and increasing $\mathrm{thr}_{PC}$ from 0.5 to 0.65 with 0.05 as an increment.

**References:**

Wen, X., Zhang, H., Li, G., Liu, M., Yin, W., Lin, W., et al. (2019). First-year development of modules and hubs in infant brain functional networks. *Neuroimage* 185**,** 222-235.
